# Supplementary material for: Redox nanomedicine ameliorates chronic kidney disease (CKD) by mitochondrial reconditioning in mice
Source: Commun Biol. 2021 Aug 26;4:1013. doi: 10.1038/s42003-021-02546-8 (PMC8390471; doi:10.1038/s42003-021-02546-8)
Supplement: Supplementary file 2 — Supplementary Information [file 42003_2021_2546_MOESM2_ESM.pdf]

Supplementary Information

Redox Nanomedicine Ameliorates Chronic Kidney Disease (CKD) by Mitochondrial Reconditioning

Aniruddha Adhikari[1], Susmita Mondal[1], Tanima Chatterjee[2], Monojit Das[3,4], Pritam Biswas[5], Ria Ghosh[2], Soumendra Darbar[6], Hussain Alessa[7], Jalal T. Althakafy[7], Ali Sayqal[7], Saleh A. Ahmed[7,8], Anjan Kumar Das[9], Maitree Bhattacharyya[2], Samir Kumar Pal[1,3,*]

[1]Department of Chemical, Biological and Macromolecular Sciences,
S. N. Bose National Centre for Basic Sciences,
Block JD, Sector 3, Salt Lake, Kolkata-700106, India

[2]Department of Biochemistry,
University of Calcutta
35, Ballygunge Circular Road, Kolkata-700019, India

[3]Department of Zoology,
Uluberia College, University of Calcutta,
Uluberia, Howrah-711315, India

[4]Department of Zoology,
Vidyasagar University,
Rangamati, Midnapore-721102, India

[5]Department of Microbiology,
St. Xavier's College,
30, Mother Teresa Sarani, Kolkata-700016, India

[6]Research & Development Division,
Dey's Medical Stores (Mfg.) Ltd,
62, Bondel Road, Ballygunge, Kolkata-700019, India

[7]Department of Chemistry, Faculty of Applied Sciences,
Umm Al-Qura University,
21955 Makkah, Saudi Arabia

[8]Chemistry Department, Faculty of Science,
Assiut University,
71516 Assiut, Egypt

[9]Department of Pathology,
Calcutta National Medical College and Hospital,
32, Gorachand Rd, Beniapukur, Kolkata-700014, India

***Corresponding Author:** **Prof. (Dr.) Samir Kumar Pal**
E-mail: skpal@bose.res.in
Phone: (+91)33 2335 5706-08
Fax: (+91)33 2335 3477

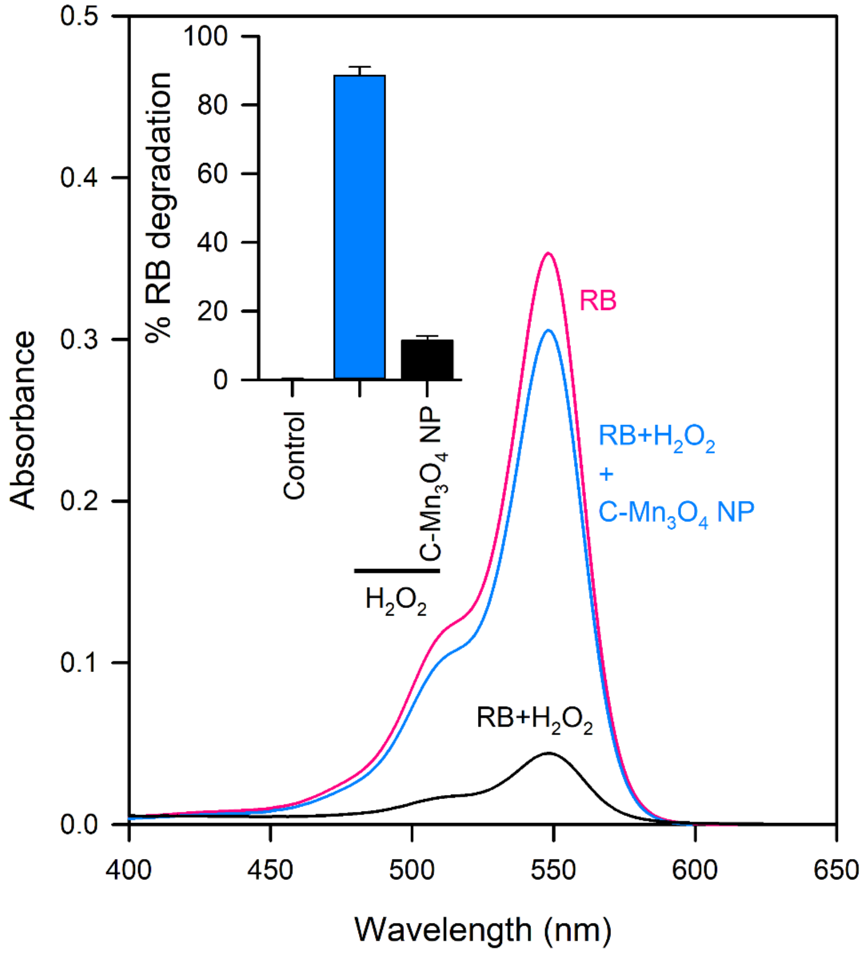

Supplementary Figure S1. H_2O_2 scavenging activity of C-Mn_3O_4 NPs using Rose Bengal (RB) assay. The RB undergoes oxidative degradation upon interaction with H_2O_2 as indicated in significantly decreased absorbance spectra. In presence of C-Mn_3O_4 NPs, H_2O_2 cannot degrade RB due to radical scavenging activity of the NPs. The inset shows the percentage of RB degradation by H_2O_2 in absence and presence of C-Mn_3O_4 NPs. Data are expressed as Mean ± SD.

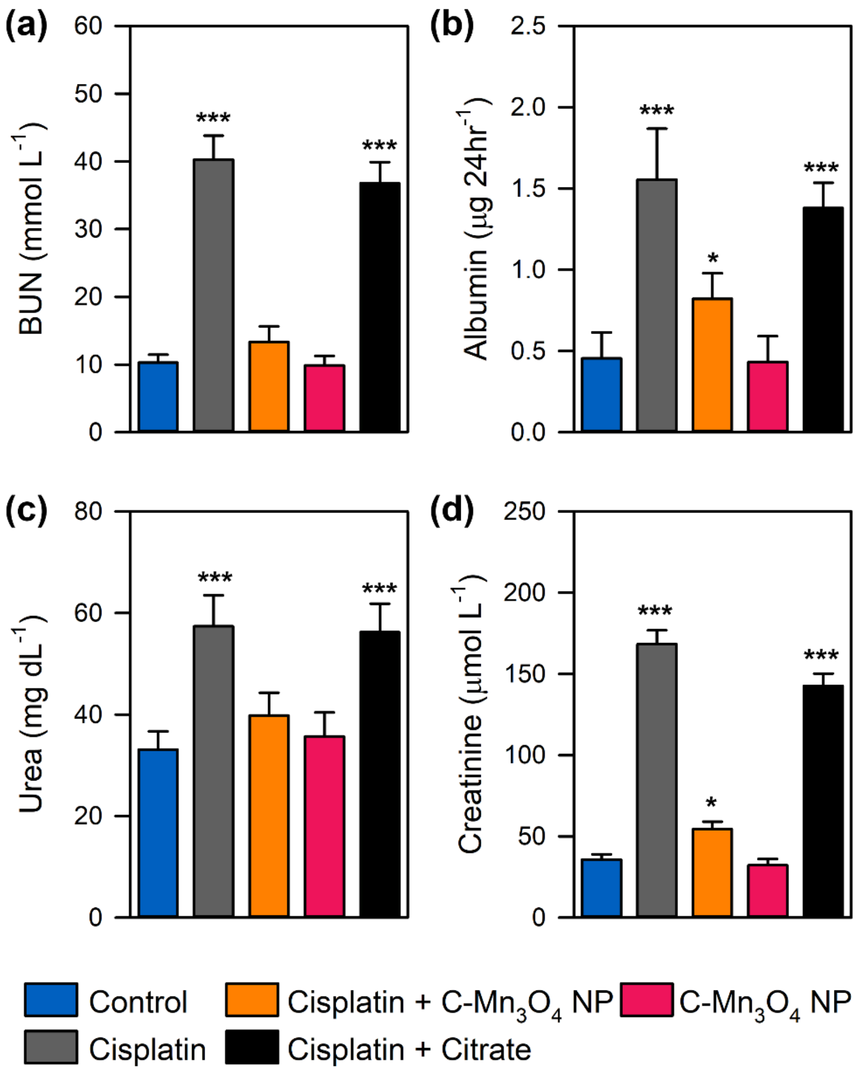

Supplementary Figure S2. Efficacy of C-Mn_3O_4 NPs in reversal of CKD in animal model. (a) Blood urea nitrogen (BUN) content. (b) Urinary albumin excretion as an indicator of albuminuria, hallmark of CKD. (c) Serum urea concentration. (d) Serum creatinine level. Note, treatment with citrate (the ligand) could not reduce the cisplatin induced nephrotoxicity. So, we left the treatment group from further downstream studies.

Data are expressed as Mean ± SD. *N*=6. *, **, *** Values differ significantly from control group (without treatment) (***$p < 0.001$; **$p < 0.01$; *$p < 0.05$).
