# Supplementary material for: Redox nanomedicine ameliorates chronic kidney disease (CKD) by mitochondrial reconditioning in mice
Source: Commun Biol. 2021 Aug 26;4:1013. doi: 10.1038/s42003-021-02546-8 (PMC8390471; doi:10.1038/s42003-021-02546-8)
Supplement: Supplementary file 3 — Reporting Summary [file 42003_2021_2546_MOESM3_ESM.pdf]

Corresponding author(s): Samir Kumar Pal

Last updated by author(s): Jul 14, 2021

Reporting Summary

Nature Research wishes to improve the reproducibility of the work that we publish. This form provides structure for consistency and transparency in reporting. For further information on Nature Research policies, see our Editorial Policies and the Editorial Policy Checklist.

Statistics

For all statistical analyses, confirm that the following items are present in the figure legend, table legend, main text, or Methods section.

n/a	Confirmed	
☐	☒	The exact sample size (*n*) for each experimental group/condition, given as a discrete number and unit of measurement
☐	☒	A statement on whether measurements were taken from distinct samples or whether the same sample was measured repeatedly
☐	☒	The statistical test(s) used AND whether they are one- or two-sided<br>*Only common tests should be described solely by name; describe more complex techniques in the Methods section.*
☒	☐	A description of all covariates tested
☐	☒	A description of any assumptions or corrections, such as tests of normality and adjustment for multiple comparisons
☐	☒	A full description of the statistical parameters including central tendency (e.g. means) or other basic estimates (e.g. regression coefficient) AND variation (e.g. standard deviation) or associated estimates of uncertainty (e.g. confidence intervals)
☐	☒	For null hypothesis testing, the test statistic (e.g. *F*, *t*, *r*) with confidence intervals, effect sizes, degrees of freedom and *P* value noted<br>*Give P values as exact values whenever suitable.*
☒	☐	For Bayesian analysis, information on the choice of priors and Markov chain Monte Carlo settings
☒	☐	For hierarchical and complex designs, identification of the appropriate level for tests and full reporting of outcomes
☒	☐	Estimates of effect sizes (e.g. Cohen's *d*, Pearson's *r*), indicating how they were calculated

Our web collection on statistics for biologists contains articles on many of the points above.

Software and code

Policy information about availability of computer code

Data collection	Data were collected using the proprietary softwares provided by the manufacturers of respective instruments detailed in the methods section of the manuscript.
Data analysis	ANOVA- GraphPad Prism v8.00; Image analysis- ImageJ; DNA gel analysis- GeneSys; Assembly of images- Adobe Photoshop CC and Illustrator CC; Survival analysis- SigmaPlot v14.00; Data fitting- Origin Pro v8.5; Plotting- GraphPad Prism v8.00 and SigmaPlot v14.00.

For manuscripts utilizing custom algorithms or software that are central to the research but not yet described in published literature, software must be made available to editors and reviewers. We strongly encourage code deposition in a community repository (e.g. GitHub). See the Nature Research guidelines for submitting code & software for further information.

Data

Policy information about availability of data

All manuscripts must include a data availability statement. This statement should provide the following information, where applicable:

- Accession codes, unique identifiers, or web links for publicly available datasets
- A list of figures that have associated raw data
- A description of any restrictions on data availability

All required data are provided in the manuscript. The datasets generated and analyzed during this study to support the findings are available in a DOI-minting online open access repository, figshare, with the identifier (DOI: 10.6084/m9.figshare.14995122).

Field-specific reporting

Please select the one below that is the best fit for your research. If you are not sure, read the appropriate sections before making your selection.

- [x] Life sciences
- [ ] Behavioural & social sciences
- [ ] Ecological, evolutionary & environmental sciences

For a reference copy of the document with all sections, see nature.com/documents/nr-reporting-summary-flat.pdf

Life sciences study design

All studies must disclose on these points even when the disclosure is negative.

Sample size	The minimum number of samples required (N=6) to get a statistically significant result in biochemical and molecular studies was chosen based on previously reported similar studies (Nat Med 11(8):867-874, 2005; J Am Soc Nephrol 22:1041-1052, 2011; Diabetes 63:1366-1380, 2014; J Am Soc Nephrol 27:3331-3344, 2016).<br>Number of animals in each group (N=16) was determined based on the minimum number of animals required to obtain sufficient amount of samples (e.g., blood, organ homogenate etc.) to perform biochemical and molecular studies, and recommendation of the Institutional Animal Ethics Committee (IAEC).
Data exclusions	No data were excluded.
Replication	All the experimental findings were reliably reproduced by replicating the experiments in both mice and cellular systems.
Randomization	Experimental and control mice were randomly chosen from littermates having the correct genotype. Random samples from two experiments were selected for biochemical, histological and molecular analysis. Experiments were not randomized.
Blinding	No blinding was used during experiments or data analysis. Only, the clinical histopathologist was completely blinded to the samples (treatment groups) during histopathological observations and scoring.

Reporting for specific materials, systems and methods

We require information from authors about some types of materials, experimental systems and methods used in many studies. Here, indicate whether each material, system or method listed is relevant to your study. If you are not sure if a list item applies to your research, read the appropriate section before selecting a response.

Materials & experimental systems

n/a	Involved in the study	
[ ]	[x]	Antibodies
[ ]	[x]	Eukaryotic cell lines
[x]	[ ]	Palaeontology and archaeology
[ ]	[x]	Animals and other organisms
[x]	[ ]	Human research participants
[x]	[ ]	Clinical data
[x]	[ ]	Dual use research of concern

Methods

n/a	Involved in the study	
[x]	[ ]	ChIP-seq
[ ]	[x]	Flow cytometry
[x]	[ ]	MRI-based neuroimaging

Antibodies

Antibodies used	Primary antibody in IHC: Rat anti-mouse CD68 antibody (Santa Cruz Biotechnology, India). This monoclonal antibody is cross-reactive to CD-68 of mouse, rat and human origin.<br>Secondary antibody in IHC: HRP-conjugated rabbit anti-rat secondary antibody (Santa Cruz Biotechnology, India).
Validation	Both primary and secondary antibodies are well-validated in the literature (Primary: Nat Biomed Eng 2:810–821, 2018; J Clin Invest 129(4):1684-1698, 2019 and Secondary: Sci Adv 7(26): eabg2517, 2021; Cell Rep 34: 108756, 2021).

Eukaryotic cell lines

Policy information about cell lines

Cell line source(s)	Human Embryonic Kidney Cells (HEK 293) were obtained from National Centre for Cell Sciences (NCCS), India.
Authentication	Authentication was done by NCCS as per internal quality control procedure. No further authentication was performed.
Mycoplasma contamination	Mycoplasma contamination was checked using commercially available test kit (Himedia, India) as per manufacturer's instruction.

Commonly misidentified lines (See ICLAC register) | n/a

Animals and other organisms

Policy information about studies involving animals; ARRIVE guidelines recommended for reporting animal research

Laboratory animals: Healthy 8-10 weeks old non-diabetic C57BL/6j mice of both sexes (50% male and 50% female) were used.

Wild animals: No wild animal was used

Field-collected samples: n/a

Ethics oversight: The animal studies were conducted at Uluberia College, and approved by Institutional Animal Ethics Committee (IAEC), Approval No.-05/S/UC-IAEC/01/2019. The guideline of Committee for the Purpose of Control and Supervision of Experiments on Animals (CPCSEA), Govt. of India, was followed.

Note that full information on the approval of the study protocol must also be provided in the manuscript.

Flow Cytometry

Plots

Confirm that:

- [x] The axis labels state the marker and fluorochrome used (e.g. CD4-FITC).
- [x] The axis scales are clearly visible. Include numbers along axes only for bottom left plot of group (a 'group' is an analysis of identical markers).
- [x] All plots are contour plots with outliers or pseudocolor plots.
- [x] A numerical value for number of cells or percentage (with statistics) is provided.

Methodology

Sample preparation: For flow cytometry, after treatment cells were trypsinized, washed with 1X PBS and stained with DCFH2-DA (15 µM; Sigma, USA) for 10 mins at 30°C in dark.

Instrument: FACS Verse, Beckton Dickinson (BD), San Jose, USA.

Software: BD FACSuite™ software.

Cell population abundance: Ten thousand events were analyzed by flow cytometry and the respective mean fluorescence intensity (in FL1 channel, set with a 530/30 nm bandpass filter) values were correlated with the ROS levels.

Gating strategy: n/a

- [ ] Tick this box to confirm that a figure exemplifying the gating strategy is provided in the Supplementary Information.
